# Bellevue Hospital

**Published:** 1854-03

**Authors:** 


*Bellevue Hospital.*— Drs. Willard Parker and Wm. H. Van Buren have resigned their respective situations as Surgeons to Bellevue Hospital, which they have held much to the credit of that Institution; and Drs. Lewis A. Sayre and John J. Crane, have been duly appointed to the vacancies thus created.

